# Report of Committee on Army Legislation1Thirty-seventh Annual Meeting of A. V. M. A., Detroit, September, 1900.

**Published:** 1900-09

**Authors:** D. E. Salmon, R. S. Huidekoper, W. Horace Hoskins, A. W. Clement, M. Stalker

**Affiliations:** Chairman


### REPORT OF COMMITTEE ON ARMY LEGISLATION.[1]

YOUR committee which was appointed to urge suitable legislation for the improvement of the veterinary service in the United States Army, and for the recognition of veterinarians in that service as representatives of a learned profession, would respectfully report that they have labored diligently to secure the desired objects, and that a satisfactory measure is now pending.

It is not necessary to go into details concerning the opposition which has been experienced. The opposition which has been most determined and most difficult to overcome has originated in the very department whose service we have been endeavoring to strengthen and perfect—the one department which would be benefited by this legislation. The cause of this opposition appears to be principally the old narrow-minded and un-American prejudice existing against veterinarians among certain of the army officers, principally the lieutenants. There is undoubtedly a feeling among this order of gentlemen that when a man becomes a lieutenant he belongs in a class by himself; that he is superior to and made of finer clay than ordinary mortals, and subject to contamination if he associates with them on equal terms. He regards himself, like the Chinese sovereign, as the Son of Heaven, to whom all should

[1] Thirty-seventh Annual Meeting of A. V. M. A., Detroit, September, 1900.

bow down and render obeisance. He expects to be gazed at from afar, admired, feared; but he cannot endure the idea of equality, familiarity, least of all with veterinarians. According to his philosophy, if one son chooses for his profession the destruction of his fellow-man, and in due time becomes a second lieutenant, and another son in the same family chooses as his lifework the amelioration of suffering among God's dumb creatures, the latter, though equally refined, equally educated, equally intelligent, is unfit to associate with the former.

This absurd self-conceit, arrogance, and bumptiousness, notwithstanding that it has done much to render the army unpopular with the masses of our people, and is doing its part toward preventing an increase commensurate with the development and needs of the country, has had its influence in the War Department and in Congress, but can never secure the support of a majority of the representatives of the American people. The importance of an organized and competent veterinary service in the army is becoming more apparent every year, and every time a Senator asserts that a captain knows more about a horse than does a veterinarian he inspires his listeners to ask mentally, if not orally, Why, on the same principle, does not a captain know more about a man than does a surgeon? Why does he not know more about engineering than does an engineer? This is an age of specialties, and a captain cannot be his own surgeon, his own engineer, his own veterinarian, and at the same time attend to his other duties. This should be self-evident to the least experienced person. Fortunately these petty and antiquated arguments, though repeated *ad nauseam*, have not had much influence in the hall of Congress. It is no longer the arguments which must be answered; it is the personal appeals which must be counteracted.

A bill has passed the Senate providing for a properly organized veterinary corps which would supply veterinary service for the cavalry, the artillery, the transportation department, and the military schools. This bill has gone to the House of Representatives, and is now in the Committee on Military Affairs. While the Secretary of War does not favor the legislation establishing a veterinary corps, he has recommended that the veterinarians in the cavalry regiments be given the rank of second lieutenants. This is a distinct advance for the War Department, and is extremely encouraging for all who have worked these many years to secure some amelioration of the army veterinarian's condition.

This legislation has therefore reached the critical period. There has never been a time when the conditions were more encouraging for success. There has never been a time when there was a prospect of accomplishing so much. There has never been a time when the same amount of work would be as effective as it will during the next six months.

Your committee, created to further the cause of army veterinary legislation, therefore, appeals to this Association and to the veterinary profession throughout the country to make a supreme effort now, while the prospects are so good, and to leave nothing undone which will contribute to success. Every one of the 8000 veterinarians in the country should feel a personal interest in this legislation. It secures the full recognition of the Government for the veterinary profession. It raises the standard of the veterinary profession in this country to the same level which it has reached in other civilized countries. It provides an army veterinary service of which we may be proud, and to which every graduate might aspire with the feeling that he has chosen an honorable and meritorious life-work. It perfects our army organization and gives the Government animals the same skilful and merciful treatment which has long since been accorded them by other intelligent and humane nations.

This is a part of the great cause for which every true veterinarian has studied and labored, of which he has pondered by day and dreamed at night, for which he has lived and for which he is willing to die. Therefore let every individual veterinarian say to himself: This is the opportunity of *my* life; *I* will do *personally* everything in my power to secure the success of this effort *in the cause of humanity*, this effort to strengthen the army of *my country*, this effort to increase the standing and reputation of *my profession.*

D. E. SALMON, *Chairman*,
R. S. HUIDEKOPER,
W. HORACE HOSKINS,
A. W. CLEMENT,
M. STALKER.

